# *De novo* production of the monoterpenoid geranic acid by metabolically engineered *Pseudomonas putida*

**DOI:** 10.1186/s12934-014-0170-8

**Published:** 2014-12-04

**Authors:** Jia Mi, Daniela Becher, Patrice Lubuta, Sarah Dany, Kerstin Tusch, Hendrik Schewe, Markus Buchhaupt, Jens Schrader

**Affiliations:** DECHEMA Research Institute, Frankfurt am Main, Germany

**Keywords:** *Pseudomonas putida*, *de novo*, DSM12264, Microbial cell factory, Monoterpene, Monoterpenoid, Mevalonate pathway, Metabolic engineering, Geranic acid, Geraniol

## Abstract

**Background:**

Production of monoterpenoids as valuable chemicals using recombinant microbes is a growing field of interest. Unfortunately, antimicrobial activity of most monoterpenoids hampers a wide application of microorganisms for their production. Strains of *Pseudomonas putida*, a fast growing and metabolically versatile bacterium, often show an outstanding high tolerance towards organic solvents and other toxic compounds. Therefore, *Pseudomonas putida* constitutes an attractive alternative host in comparison to conventionally used microorganisms. Here, metabolic engineering of solvent tolerant *Pseudomonas putida* as a novel microbial cell factory for *de novo* production of monoterpenoids is reported for the first time, exemplified by geranic acid production from glycerol as carbon source. The monoterpenoic acid is an attractive compound for application in the flavor, fragrance, cosmetics and agro industries.

**Results:**

A comparison between *Escherichia coli*, *Saccharomyces cerevisiae* and *Pseudomonas putida* concerning the ability to grow in the presence of geranic acid revealed that the pseudomonad bears a superior resilience compared to the conventionally used microbes. Moreover, *Pseudomonas putida* DSM 12264 wildtype strain efficiently oxidized externally added geraniol to geranic acid with no further degradation. Omitting external dosage of geraniol but functionally expressing geraniol synthase (GES) from *Ocimum basilicum*, a first proof-of-concept for *de novo* biosynthesis of 1.35 mg/L geranic acid in *P. putida* DSM 12264 was achieved. Doubling the amount of glycerol resulted in twice the amount of product. Co-expression of the six genes of the mevalonate pathway from *Myxococcus xanthus* to establish flux from acetyl-CoA to the universal terpenoid precursor isopentenylpyrophosphate yielded 36 mg/L geranic acid in shake flask experiments. In the bioreactor, the recombinant strain produced 193 mg/L of geranic acid under fed-batch conditions within 48 h.

**Conclusion:**

Metabolic engineering turned *Pseudomonas putida* DSM 12264, a versatile monoterpenoid oxidation biocatalyst, into an efficient microbial cell factory for *de novo* geranic acid production. Improvements by metabolic and process engineering are expected to further increase the product concentration. To the best of the authors’ knowledge, this is the first example of a *de novo* production of a monoterpenoid with *Pseudomonas putida* and of a microbial monoterpenoic acid synthesis in general.

**Electronic supplementary material:**

The online version of this article (doi:10.1186/s12934-014-0170-8) contains supplementary material, which is available to authorized users.

## Background

Terpenoids constitute the most diverse group of natural products with more than 40,000 structurally different molecules identified in nature [1], of which more than 1,000 compounds belong to the subclass of monoterpenoids [2]. Terpenoids are widely applied as flavor and fragrance compounds, colorants, nutraceuticals, pharmaceuticals, agrichemicals etc. [1]. Since these biomolecules, especially their oxyfunctionalized derivatives, are often naturally produced in only small amounts, approaches for their production others than isolation from natural raw materials are very much sought. Since chemical synthesis often starts from fossil raw materials and can be costly, especially for stereo- and/or regioselectively oxyfunctionalized terpenoids, biotechnological production starting from cheap renewable substrates offers a highly attractive alternative. In their pioneering work, J.D. Keasling and co-workers comprehensively engineered *Escherichia coli* and *Saccharomyces cerevisiae* towards microbial cell factories for the production of the sesquiterpenoids amorpha-4,11-diene and artemisinic acid [3–6]. Nowadays, high concentrations regarding the production of secondary metabolites of up to the grams per liter range are reported for sesquiterpenoids already in shake flask cultures [7–9], but monoterpenoid production using similarly engineered microbial hosts as platform strains is usually still limited to product titers in the lower milligrams per liter range [10,11]. The toxicity of monoterpenoids is referred to as the main bottleneck hampering their efficient microbial production or biotransformation [12,13]. It was shown that monoterpenoids, such as thymol, menthol, linalyl acetate, α-pinene and β-pinene, preferentially intercalate into the cell membrane resulting in an increase of membrane fluidity which eventually leads to loss of vital membrane functions and cell death [14–16]. For baker’s yeast, the cell wall instead of the membrane was recently shown to be the site of the destructive action of externally added limonene [17]. *Pseudomonas putida*, a Gram-negative γ-proteobacterium, possesses the outstanding ability to tolerate high concentrations of different hydrocarbons, though great differences in the degree of resilience occur among different strains. Several mechanisms to counteract organic solvent toxicity are described for *P. putida* such as reinforcing the membrane phospholipid bilayer by *cis*-to-*trans* isomerization of unsaturated fatty acids, increasing the ratio of saturated-to-unsaturated fatty acids, and active export of toxic compounds via efflux pumps [18,19]. A couple of industrial processes with *P. putida* have already been established [20] and more wildtype and recombinant *P. putida* strains have been recently described for potential industrial *de novo* production of toxic aromatic compounds such as phenol [21] and *p*-hydroxybenzoate [22] or for biotransformation of toxic precursors such as toluene to *o*-cresol [23] or the monoterpenoid limonene to perillic acid [24]. This illustrates the potential of this species as an alternative host for biotechnological applications, especially if toxic substrates or products are dealt with. However, *de novo* monoterpenoid production with *P. putida* has not been reported until the present work.

The aim of our present study was to investigate the potential of a solvent tolerant *P. putida* strain to be engineered as a microbial cell factory for *de novo* production of monoterpenoids. We chose *P. putida* DSM 12264 and geranic acid as our model strain and product, respectively, for three reasons: i) we knew from our previous work that this strain is highly robust in the presence of limonene even up to volume shares which form a distinct separate organic phase in the bioreactor [24], ii) previous work with this strain [25] as well as unpublished own pre-investigations revealed that the wildtype strain is also resilient against other monoterpenoids and it can convert the monoterpene alcohol geraniol to geranic acid, and iii) geranic acid has great potential for different industrially relevant applications. Geranic acid can be used as a perfuming agent [26] and is an important building block for the production of natural flavor esters [27]. Moreover, it shows strong antifungal properties against two main phytopathogens of corn, *Colletotrichum graminicola* and *Fusarium graminearum*, and was therefore produced in a transgenic maize plant to improve resistance against the fungal attack [28]. Furthermore, this monoterpenoid is known to inhibit tyrosinase, a key enzyme of melanogenesis in mammalian cells [29]. Lately, Choi showed that geranic acid has depigmenting properties in melanocytes combined with low cell toxicity which makes this compound attractive as skin depigmentation agent [30].

Here, we introduced genes for a truncated geraniol synthase (GES) from sweet basil *Ocimum basilicum* [31] and the complete mevalonate (MVA) pathway from the Gram-negative bacterium *Myxococcus xanthus* into *P. putida* DSM 12264. The recombinant strain was able to produce geranic acid without significant terpenoid by-product formation and, due to its monoterpenoid robustness, may serve as a platform strain for monoterpenoid production in the future.

## Results

### Suitability of *P. putida* as a host for geranic acid production

**Figure 1:**
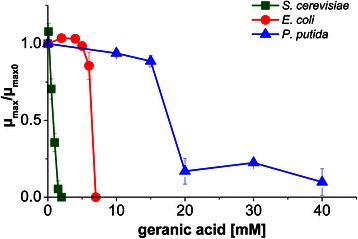
**Growth inhibition of** ***S. cerevisiae*** **,** ***E. coli*** **and** ***P. putida*** **by geranic acid.** Indicated are the ratios of the maximum growth rate at given geranic acid concentrations (μ_max_) to the maximum growth rate without monoterpenoid (μ_max0_) at different geranic acid concentrations for *S. cerevisiae*, *E. coli* and *P. putida*. Cells were grown in screw cap shake flasks in complex medium and different geranic acid amounts were added at the beginning of the exponential phase. Samples were taken for OD measurements to calculate μ_max_. Mean values of two independent experiments, indicated by error bars, are given.

To verify the expected advantage of *P. putida* for monoterpenoid production compared with conventional host strains, i.e. an assumed superior product tolerance, growth assays with *P. putida* DSM 12264, *E. coli* DH5α and *S. cerevisiae* CEN.PK2-1C in the presence of geranic acid were performed (Figure 1). According to Brennan and colleagues, shake flasks with screw caps were used for growth experiments to avoid monoterpenoid evaporation [32]. Growth of *S. cerevisiae* strongly decreased when geranic acid concentrations were increased and was completely inhibited at 2 mM geranic acid, whereas *E. coli* growth became significantly affected by geranic acid starting from concentrations of 5 mM and was completely inhibited at a concentration of 7 mM. For *P. putida*, cell growth was distinctly impaired at 20 mM but did not cease even at the highest geranic acid concentration tested, which was 40 mM.

To exclude biodegradation and bioconversion of the desired product by *P. putida* DSM 12264, utilization of geranic acid by this bacterium and product stability were examined. Neither biodegradation nor bioconversion of geranic acid was observed (data not shown). Furthermore, *P. putida* KT2440 was also tested for geraniol oxidation, since this strain is far better described in literature than *P. putida* DSM 12264 [33]. This strain showed a geranic acid tolerance similar to *P. putida* DSM 12264 and no further metabolization of this monoterpenoic acid could be observed as well (data not shown).

### Biotransformation of geraniol to geranic acid by wildtype *P. putida* DSM 12264

**Figure 2:**
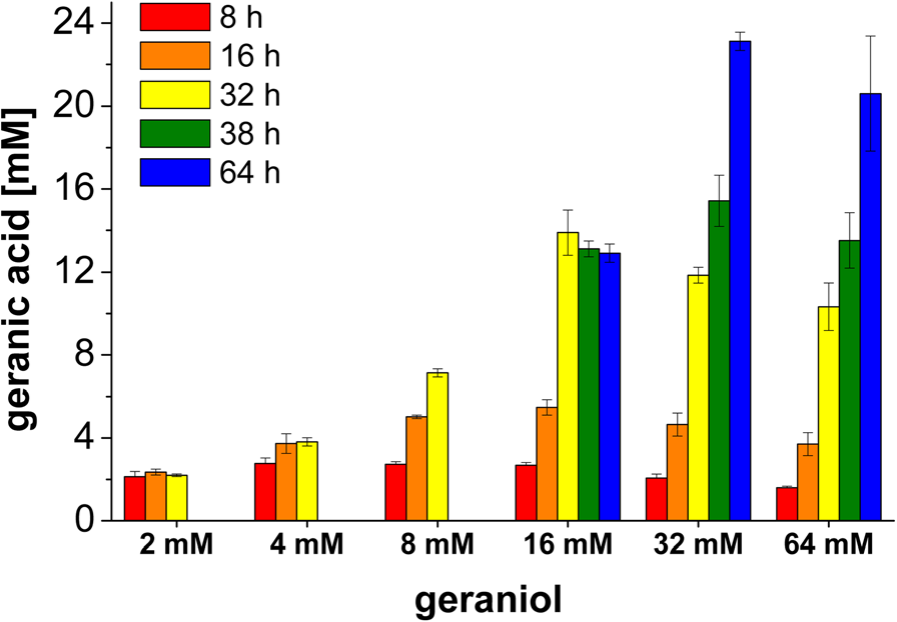
**Whole cell biotransformation of geraniol by** ***P. putida*** **DSM 12264 wildtype strain.** Indicated are the concentrations of produced geranic acid at different time points by whole cell biotransformation of geraniol by *P. putida* DSM 12264 wildtype. Different amounts of geraniol were added to growing cells in E2 medium (containing 10% LB and 30 mg/L kanamycin, 4.6 g/L glycerol) and geranic acid concentration was quantified after 8, 16, 32, 38 and 64 h. For 2, 4 and 8 mM geraniol, respectively, the last sample was taken after 32 h due to complete geraniol depletion and maximum product concentration. Experiments were run in triplicates. For detailed statistical analysis, please see Additional file 1.

Pseudomonads are known to have strong oxidation capabilities towards different hydrocarbons. Besides the oxidation of monoterpene hydrocarbons, such as limonene or the pinenes, *Pseudomonas s*pecies have also been shown to oxidize monoterpene aldehydes, such as citral or citronellal, and monoterpene alcohols, such as linalool or nerol [34]. To demonstrate the natural capacity of geraniol oxidation in cells of *P. putida* DSM 12264, different amounts of geraniol were added to growing cells in E2 medium and resulting geranic acid concentrations were determined at different time points within 64 h (Figure 2). Up to 16 mM geraniol were nearly completely oxidized within the tested time period. With an initial substrate concentration of 32 mM, a maximum product concentration of 23 mM was obtained. Furthermore, the oxidation rate decreased at 32 mM and 64 mM (for t =8 h, geranic acid concentrations were significantly (p <0.05, Tukey’s HSD) lower than at 4 mM, 8 mM and 16 mM geraniol). For more information about the statistical analysis, please see Additional file 1. Cell growth slowed down at 8 mM geraniol and was distinctly impaired at 16 mM (data not shown). Cell lysis was observed at 32 mM and higher concentrations after 36 h (data not shown), while oxidation rate decreased correspondingly.

These data illustrate the high geraniol oxidation capacity of *P. putida* DSM 12264 with a maximum geranic acid production rate over the first 32 h of ~0.43 mM/h. In contrast, an about 50% lower product formation rate and a maximum product concentration of 5 mM geranic acid were observed for *P. putida* KT2440 (data not shown).

### Geranic acid production of *P. putida* DSM 12264 expressing GES

**Figure 3:**
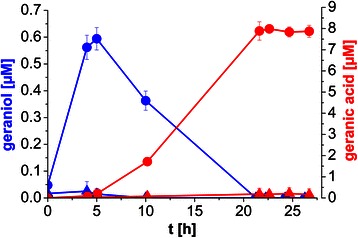
**Production of geraniol and geranic acid by** ***P. putida*** **DSM 12264 expressing GES.** Indicated are the concentrations of *de novo* produced geraniol (blue) and geranic acid (red) over time using *P. putida* DSM 12264 expressing GES. Cells were grown in shake flasks in E2 medium (containing 10% LB and 30 mg/L kanamycin, 4.6 g/L glycerol) at 30°C and GES expression was induced 3 h after inoculation with 0.2% (w/v) *L*-rhamnose. Triangles indicate *P. putida* DSM 12264 with empty plasmid, circles indicate *P. putida* DSM 12264 pMiS1-ges. Geraniol and geranic acid concentrations were determined via HPLC over time. Mean values of two independent experiments, indicated by error bars, are given. For detailed statistical analysis, please see Additional file 3.

To implement *de novo* geraniol production in *P. putida*, the gene of geraniol synthase (GES) from *O. basilicum,* encoding the enzyme converting the cellular terpenoid biosynthesis intermediate geranyl pyrophosphate (GPP) to geraniol*,* was introduced via plasmid pMiS1 (Additional file 2). Recombinant cells were cultivated in E2 medium (containing 10% LB) for 3 h, before gene expression was induced by addition of 0.2% (w/v) *L*-rhamnose (t = 0, Figure 3). A maximum concentration of 0.6 μM geraniol was measured after 5 h followed by continuous depletion, while geranic acid accumulated simultaneously, ending up with a maximum concentration of 8 μM (ca. 1.3 mg/L) after about 21 h. No geraniol and geranic acid was detected in cultures of *P. putida* DSM 12264 containing an empty vector (for statistical analysis, see Additional file 3).

### Geranic acid production in *P. putida* DSM 12264 coexpressing GES and MVA pathway

The efficiency of microbial terpenoid production is limited by the flux from intermediates of the central metabolism, i.e. acetyl-CoA (MVA pathway) or pyruvate and glyceraldehyde-3-phosphate (2-C-methylerythritol-4-phosphate (MEP) pathway) towards the terpene synthase substrates geranyl pyrophosphate (GPP), farnesyl pyrophosphate (FPP) or geranylgeranyl pyrophosphate (GGPP). This has already been overcome by different approaches, e.g. by overexpression of genes of the terpenoid biosynthesis or the elimination of feedback inhibition mechanisms [35]. The prime example of synthetic biology for microbial terpenoid production is the successful heterologous expression of the MVA pathway in *E. coli* increasing intracellular FPP concentration which greatly improved the yield of sesquiterpene amorpha-4,11-diene [3] and laid the foundation for a series of step-wise further strain improvements.

**Figure 4:**
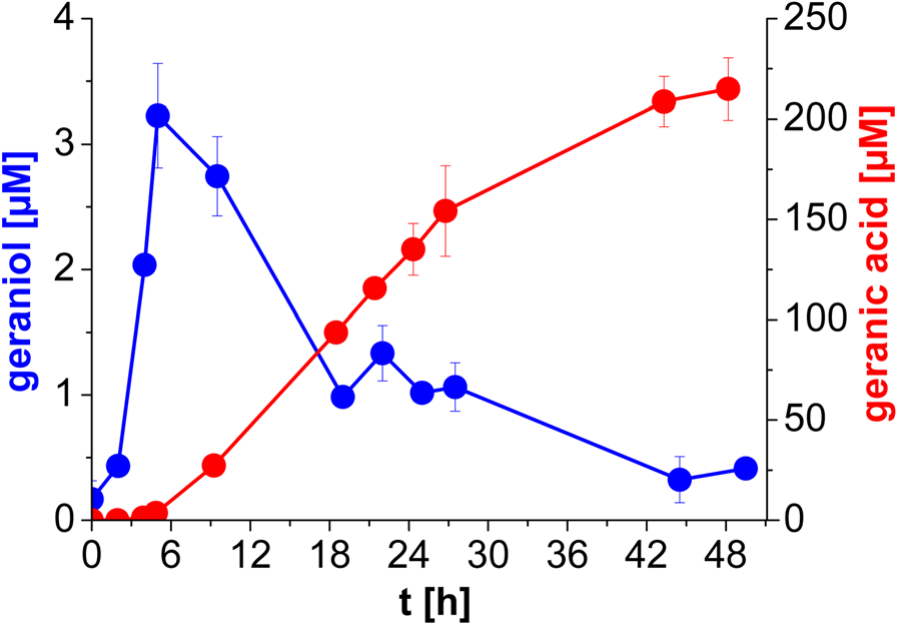
**Production of geraniol and geranic acid by** ***P. putida*** **DSM 12264 expressing GES and MVA.** Indicated are the concentrations of *de novo* produced geraniol (blue) and geranic acid (red) over time using *P. putida* DSM 12264 expressing GES and MVA. Cells were grown in shake flasks in E2 medium (containing 10% LB, 30 mg/L kanamycin and 9.2 g/L glycerol) at 30°C and heterologous gene expression was induced 3 h after inoculation with 0.2% (w/v) *L*-rhamnose. Geraniol and geranic acid concentrations were determined via HPLC over time. Mean values of two independent experiments, indicated by error bars, are given. For detailed statistical analysis, please see Additional file 4.

Following this concept to increase carbon flux towards terpenoid synthesis, 6 genes encoding the MVA pathway of *M. xanthus* leading from acetyl-CoA to isopentenyl pyrophosphate (IPP) and dimethylallyl pyrophosphate (DMAPP) together with the geraniol synthase gene from *O. basilicum* were introduced into *P. putida* DSM 12264 in the present work. In contrast to previously shown experiments, twice the amount of glycercol (9.2 g/L instead of 4.6 g/L) was used for the medium to prevent growth-limiting effects. The geraniol production/depletion and concurrent geranic acid production profiles obtained (Figure 4) look similar to the profiles of the strain lacking the MVA pathway (Figure 3) but with significantly higher maximum terpenoid concentrations (p <0.01, Tukey’s HSD, Additional file 4). About 3 μM of geraniol accumulated in 5 h after induction of heterologous gene expression. Geraniol concentration decreased after 5 h and was not detectable anymore after 2 days, whereas geranic acid steadily accumulated concurrently. A maximum concentration of 215 μM geranic acid was obtained after 2 days.

### Geranic acid production in the fed-batch bioreactor

**Figure 5:**
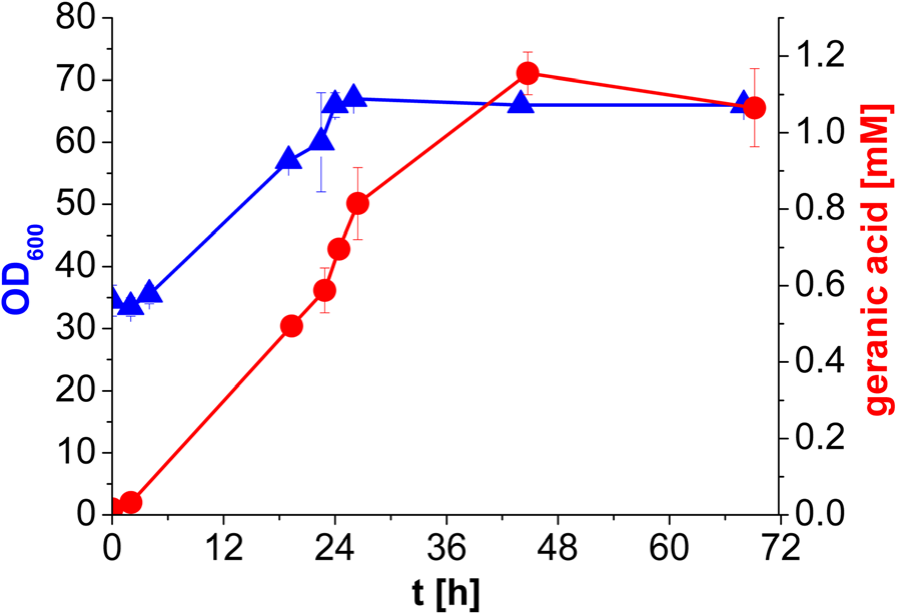
**Production of geranic acid by** ***P. putida*** **DSM 12264 expressing GES and MVA in a fed-batch bioprocess.** Indicated are the OD_600_ value (blue) and the concentration of *de novo* produced geranic acid (red) over time using *P. putida* DSM 12264 expressing GES and MVA in the bioreactor. Cells were grown in a bioreactor in E2 medium (containing 10% LB and 30 mg/L kanamycin, 4.6 g/L glycerol) for 24 h before induction of gene expression with 0.2% (w/v) *L*-rhamnose (t = 0). Geranic acid concentration was determined via HPLC over time. Mean values of two independent experiments, indicated by error bars, are given.

*P. putida* DSM 12264 coexpressing GES and the MVA pathway was tested under controlled conditions in the bioreactor. After cells were grown for 24 h, protein expression was induced (t = 0). Samples for OD_600_ measurement and HPLC analysis were taken over time for three days; the results are shown in Figure 5. Highest cell density of OD_600_ 66 was obtained after 24 h and a total of 1.15 mM geranic acid was produced after 2 days.

## Discussion

### *P. putida* DSM 12264 is a suitable microbe for geraniol to geranic acid conversion

Many *P. putida* strains are known, which are highly tolerant towards organic solvents. Since bacterial monoterpenoid toxicity is an obstacle for *E. coli* and *S. cerevisiae* as terpenoid production hosts [32,36], *P. putida* DSM 12264, known for its high monoterpenoid tolerance [24], was tested as a host for *de novo* production of geranic acid. This strain turned out to be significantly more tolerant to geranic acid than *E. coli* and *S. cerevisiae* (Figure 1). Besides, the pseudomonad is able to completely and efficiently oxidize up to 16 mM geraniol. As a consequence, inhibition of cell growth by geraniol as an intermediate is not expected to be a bottleneck during *de novo* production of geranic acid.

Some pseudomonads, such as *P. aeruginosa*, *P. citronellolis* and *P. mendocina*, are known to utilize geraniol and geranic acid as sole carbon sources whereas *P. putida* is usually not able to assimilate neither of the substances [37,38]. Oxidation of geraniol to the corresponding acid by the upper part of the acyclic terpene utilization (atu) pathway is assumed to be the inital step in geraniol degradation [39]. Furthermore, a pyrroloquinoline quinone dependent alcohol dehydrogenase (QEDH) encoded by the *exaA* gene was found to participate in geraniol oxidation in *P. aeruginosa* as well [40]. Therefore, oxidoreductases homologous to AtuB and AtuG of the atu pathway [39] or QEDH [40] are likely to be responsible for geraniol oxidation in *P. putida* DSM 12264 as well, although it cannot be excluded, whether nonspecific oxidoreductases are involved as well. These oxidoreductases are promising candidates for further optimization of the geraniol oxidation power of the strain.

The demonstration of high geranic acid tolerance combined with an efficient geraniol oxidation pathway and the inability to degrade geranic acid laid the foundation to engineer *P. putida* DSM 12264 towards a microbial cell factory for *de novo* production of geranic acid.

### *De novo* production of geranic acid by recombinant *P. putida* DSM 12264

Geraniol synthase (GES) from *O. basilicum* was shown to be functionally expressed in *E. coli* and *S. cerevisiae* and *de novo* geraniol production was already successfully demonstrated in both microbes [31,41,42]. Karst and co-workers obtained a *de novo* geraniol concentration of about 10 mg/L in yeast after optimization of GPP synthase activity to increase the GPP pool [43] and optimization of GES activity [44]. More recently, a geraniol concentration of 36 mg/L was reported for yeast after metabolic engineering of the intrinsic MVA pathway [45]. In the present work, naturally geraniol oxidizing *P. putida* DSM 12264 was chosen as host for functional expression of GES to accomplish direct *de novo* production of the desired oxidation product of geraniol, geranic acid. Glycerol was chosen as the carbon source since this compound is cheap and abundantly available as the main by-product of biodiesel production [46]. Transient accumulation of geraniol indicated fast but delayed oxidation of the monoterpene alcohol to geranic acid by endogenous enzymes. As the product was found in the supernatant without significant residual material left in the cells, product recovery in a later bioprocess will be facilitated.

Metabolic engineering of pathways leading to elevated terpenoid precursor levels is a common approach in biotechnological terpenoid production [47]. In *E. coli*, the recombinant MVA pathway from *S. cerevisiae* proved to be efficient [3,48], even though expression of some of the yeast enzymes revealed to be not balanced in *E. coli* [8,49,50]. Approaches replacing yeast genes *hmgr* and *hmgs* with their orthologues from *Staphylococcus aureus* [51,52] led to significant increases in production rates. An entirely prokaryotic MVA pathway was shown to enhance the coenzyme Q10 production in *E. coli* [53]. The bacterium *M. xanthus* possesses a complete MVA pathway where 5 of the 6 required genes are organized in an operon that eases a straightforward cloning procedure. We therefore tested the influence of the MVA pathway of *M. xanthus* on geranic acid production in *P. putida* DSM 12264 expressing GES (Figure 4). A final concentration of 215 μM (36.2 mg/L) of geranic acid was obtained after 2 days of cultivation, corresponding to a more than 10-fold increase compared to the strain without *M. xanthus* MVA pathway (p <0.01, Tukey’s HSD, Additional file 4), demonstrating the pronounced positive effect of the MVA pathway on terpenoid production in *P. putida* DSM 12264. Since the intrinsic GPP pool is assumed to be limiting as well, additional GPP synthases such as ERG20-2 from *S. cerevisiae* [54] or IDS from *Picea abies* [55] may further increase product formation. Furthermore, ribosome binding sites (RBS) of the FPP biosynthesis genes can be optimized, which led to a considerable increase in amorphadiene yield with *E. coli* [56]. Besides, homologous overexpression of the MEP pathway to enhance terpenoid precursors as an alternative route can be considered as well, since overexpression of this pathway was shown to benefit carotene production in *P. putida* KT2440 [57]. These options will be content of further work to enhance product formation.

### *De novo* production of geranic acid with *P. putida* DSM 12264 in a fed-batch bioreactor

Since *P. putida* DSM 12264 was previously shown to be an efficient whole cell biocatalyst for limonene biotransformation under process conditions due to high monoterpenoid tolerance and ease of cultivation [24], we tested the performance of the recombinant *P. putida* bearing GES and the MVA pathway in the bioreactor as well. Geranic acid concentration increased rapidly and continuously after induction. A maximum OD_600_ of 66 after 24 h and a final concentration of 1.15 mM (193 mg/L) geranic acid were obtained after 48 h, corresponding to a specific product yield of 9.7 mg/g cdw, below but in the same order of magnitude as compared with the value of 13.9 mg/g cdw obtained in the shake flask experiments. Comparable product yields between 7 and 17.6 mg/g cdw were recently obtained for limonene with different *E. coli* strains harboring the MVA pathway [58]. Significantly higher ratios of about 230 mg/g cdw of limonene and about 65 mg/g cdw of perillyl alcohol were obtained by Alonso-Gutierrez and co-workers [51] with an engineered MVA containing *E. coli* strain, assuming an average correlation of cdw [g/L] = OD_600_ [−] ∙0.46 [59]. In both experiments, a second organic phase was used for *in situ* product removal of the produced monoterpenoids. Since *P. putida* DSM 12264 is able to tolerate distinctly higher concentrations of geranic acid (see Figure 1) as those produced in the present work, inhibiting effects of the product on cell growth can be excluded. In contrast, engineered *S. cerevisiae* would have probably not been capable of producing a similar amount of product, as the yeast growth was already severely inhibited by 1 mM geranic acid (only 36% of μ_max0_, see Figure 1). *E. coli* wildtype significantly lost viability above 5 mM geranic acid, indicating that it could still be a production host for geranic acid competitive to our current data obtained with *P. putida* DSM 12264, providing that an additional heterologous expression of a geraniol-to-geranic acid pathway is feasible in *E. coli*. On the contrary, if the target molecules are not monoterpenoic acids but instead monoterpene alcohols, such as perillyl alcohol or geraniol, the pronounced oxidative capacity of *P. putida* DSM 12264 will be a disadvantage due to unwanted overoxidation as shown in a recent comparison of recombinant *E. coli* and *P. putida* for whole cell bioconversion of limonene to perillyl alcohol [60]. Identifying endogenous genes responsible for overoxidation, e.g. those for the geraniol-to-geranic acid conversion, followed by a targeted knock-out could be a solution if specific monoterpene alcohols (or ketones/aldehydes) are sought. The identification of oxidizing enzymes may also lead to a modular pathway design for *P. putida* to *de novo* synthesize monoterpenoids of a desired oxyfunctionalization degree. Here, we intentionally coupled an MVA pathway enhanced terpenoid biosynthesis with a plant monoterpene synthase and a so far unknown endogenous oxyfunctionalization pathway in *P. putida*. In pre-investigations (Becher et al., unpublished), *P. putida* DSM 12264 heterologously expressing *Mentha spicata* limonene synthase instead of *O. basilicum* geraniol synthase produced small amounts of perillic acid, as expected. However, since limonene, in contrast to geraniol, is a very cheap natural precursor abundantly available from renewable resources (by-product of the citrus processing industry) and perillic acid can be efficiently obtained in concentrations of up to 30 g/L through bioconversion of limonene as shown in our previous work [24], we focused on geranic acid in the present work. Nevertheless, this underpins the feasibility of turning *P. putida* DSM 12264 into a cell factory for *de novo* production of different monoterpenoids, especially different monoterpenoic acids. So far, the potential of *P. putida* DSM 12264 for geranic acid production has not yet been fully harnessed, as the strain is not significantly growth-inhibited at product concentrations of up to 15 mM. Therefore, there is still room for a 10-fold increase in final product concentration by metabolic engineering means without expecting a negative impact on the growth performance of the host. The maximum cell density of OD_600_ 66 corresponding to 19.8 g cdw/L obtained with *P. putida* DSM 12264 in the fed-batch process is still moderate. Thus, optimization of the bioprocess based on published protocols for high-cell density fermentations of *P. putida* [61–64] should be feasible and may lead to significant higher biomass and product concentrations. Finally, solvent tolerant bacteria such as *P. putida* are ideal microbes to be used in aqueous-organic two-phase fermentations [19] or in the presence of adsorbers [24] for *in situ* product removal, which facilitates the development of high-perfomance integrated bioprocesses.

## Conclusion

Engineering *P. putida* for *de novo* production of a monoterpenoid was shown for the first time. The solvent tolerant strain *P. putida* DSM 12264, originally used as a robust wildtype whole cell biocatalyst for production of perillic acid from limonene [24], was shown to possess a strong oxidative capacity and efficiently converted geraniol to geranic acid. Introducing MVA pathway and geraniol synthase turned *P. putida* DSM 12264 into a microbial cell factory for *de novo* production of geranic acid from the renewable carbon source glycerol. *P. putida* DSM 12264 was shown to be of superior geranic acid tolerance compared to the conventional microbial hosts *E. coli* and *S. cerevisiae*. Being tolerant against different monoterpenoids, viable even in the presence of a separate monoterpenoid phase under process conditions [24], and genetically well accessible make this strain a highly promising platform for monoterpenoid production in the future.

## Materials and methods

### Chemicals, wildtype strains and media

Geranic acid (85%), geraniol (98%) and thymol (99%) were purchased from Sigma-Aldrich (Taufkirchen, Germany). (*L*)-(+)-Rhamnose monohydrate, glycerol and hexane (≥99%) were purchased from Carl Roth (Karlsruhe, Germany).

For growth assays, *P. putida* strain DSM 12264, *S. cerevisiae* strain CEN.PK2-1C [65] and *E. coli* strain DH5α [66] were used. Each organism was grown in complex medium: terrific broth (TB) for *P. putida*, yeast medium (YM) for *S. cerevisiae* and lysogeny broth (LB) for *E. coli*. Additionally, LB was used as preculture medium for geranic acid production experiments with *P. putida*. For whole cell *de novo* syntheses, E2 medium [25] containing 10% LB was used. For PCR amplification of the MVA pathway genes, DNA of *M. xanthus* DSM 16526 was used.

### Plasmid construction and transformation

The gene encoding a GES lacking the signal peptide encoding region from *O. basilicum* was amplified from plasmid pMO5 [41]. The 6 genes of the MVA pathway, namely HMG-CoA synthase (*hmgs*), HMG-CoA reductase (*hmgr*), mevalonate kinase (*mvk*), phosphomevalonate kinase (*pmvk*), diphosphomevalonate decarboxylase (*mvd*) and isopentenyldiphosphateisomerase (*idi*), were amplified from genomic DNA of *M. xanthus*. Primers used in this study are summarized in Table 1.

**Table 1:** **Primers used in this study**

**Primer**	**Sequence (5‘ → 3‘)**	**Source**
pJeM1-MCS-f	[phos]AATTCAGGCGCTTTTTAGACTGGTCGTAATGAACCTCTAGAAGTATATTAGTTAAGTATAAGAAGGAGTTTAAACGGTACCGTCGACGAGCTCCTCGAGTGTACAGGATCCA	This work
pJeM1-MCS-r	[phos]AGCTTGGATCCTGTACACTCGAGGAGCTCGTCGACGGTACCGTTTAAACTCCTTCTTATACTTAACTAATATACTTCTAGAGGTTCATTACGACCAGTCTAAAAAGCGCCTG	This work
GES-fwd	GATC*GGTACC*ATGCCTCTAAGTTCAACTC	This work
GES-rev	GATC*GAGCTC*TTATTGAGTGAAGAAGAGG	This work
pMiS1-hmgs-f	GATC*GGATCC*AGGAGGAATAATATGAAGAAGCGCGTGGGAATC	This work
pMiS1-hmgs-r	GATC*AAGCTT* **CCTAGG**TCAGTTCCCTTCGGCGTAC	This work
pMiS1-MVA-f1	ATCT*GGAT* ***CC*** **TAGG**AGGAATAATATGGGCGACGACATCACTG	This work
pMiS1-MVA-r1	AACACCATGGC*GAGCTC*TC	This work
pMiS1-MVA-f2	GA*GAGCTC*GCCATGGTGTT	This work
pMiS1-MVA-r2	GTGCCCGTT*GAGCTC*CACCT	This work
pMiS1-MVA-f3	AGGTG*GAGCTC*AACGGGCAC	This work
pMiS1-MVA-r3	ATC*GAATTC* **AAGCTT**TCAGCTCAGCGCGCGCACC	This work

Italics and bold parts represent first and second (if available) restriction site used for cloning, respectively. pJeM1-MCS-f and pJeM1-MCS-r were 5’-phosphorylated ([phos]) and used as sense and antisense DNA strands to exchange *egfp* of pJeM1 with an insert comprising the rhamnose-inducible promoter *rhaP*_*BAD*_, a ribosome binding site and an MCS. For more details of the cloning strategy, see Additional file 2.

For gene expression in *P. putida* DSM 12264, plasmid pMiS1 was used which was derived from pJeM1 [67] by exchange of the eGFP gene with a synthetically constructed multiple cloning site sequence. Single stranded sense primer pJeM1-MCS-f and antisense strand primer pJeM1-MCS-r comprising the rhamnose-inducible promoter rhaP_BAD_, a ribosome binding site, a multiple cloning site (MCS) and 5′-phosphorylations were ordered from Sigma Aldrich. The promoter and the ribosome binding site were re-inserted with the new MCS since the original parts were cut out with *egfp* due to restriction sites limitations. After hybridization, the DNA fragment was ligated into *Eco*RI and *Hin*dIII precut pJeM1 backbone.

The *ges* gene was amplified from pMO5 [41] using primers GES-fwd and GES-rev and cloned via *Kpn*I and *Sac*I into pMiS1 to give pMiS1-ges. The MVA pathway genes of *M. xanthus* are genomically organized as a single gene (*hmgs)* and an operon containing the rest of the genes (*idi*, *hmgr*, *mvd*, *mvk*, *pmvk*). For cloning of these MVA pathway genes into pMiS1, *hmgs* was first amplified from genomic *M. xanthus* DNA using the primers pMiS1-hmgs-f (containing an upstream Shine-Dalgarno sequence) and pMiS1-hmgs-r and cloned via *Bam*HI and *Hin*dIII into pMiS1 yielding pMiS1-hmgs. Then, the operon (containing all other genes) was cloned sequentially into pUC18. Three amplifications were performed using primers pMiS1-MVA-f1, pMiS1-MVA-r1 (part1, containing an upstream Shine-Dalgarno-sequence), pMiS1-MVA-f2, pMiS1-MVA-r2 (part 2) and pMiS1-MVA-f3, pMiS1-MVA-r3 (part 3). Parts were ligated using *Bam*HI, *Sac*I and *Eco*RI as restriction sites. The new operon comprising all 5 genes was then cut from resulting pUC18-mva-op via *Avr*II and *Hin*dIII and ligated into pMiS1-hmgs to give pMiS1-mva. The *ges* gene was then cut from pMiS1-ges and ligated into pMiS1-mva using *Pme*I and *Bam*HI to give pMiS1-ges-mva. For a schematic of the cloning strategy, please see Additional file 2.

*P. putida* DSM 12264 was transformed by electroporation [68]. Recombinant cells were selected on LB agar containing 30 mg/L kanamycin.

### Growth, metabolization and bioconversion assays

The inhibitory effect of geranic acid on the growth of *E. coli*, *S. cerevisiae* or *P. putida* was investigated. To avoid terpenoid evaporation, 100 mL baffled flasks with screw caps were used for growth assays. For all microbes, 20 mL complex medium were inoculated with an optical density (OD_600_) of 0.2 from an overnight preculture and cultivated at 180 rpm shaking (2.5 cm displacement). *S. cerevisiae* and *P. putida* were cultivated at 30°C; *E. coli* was cultivated at 37°C. For each organism, a specific range of geranic acid concentrations was chosen, in which inhibiting effects occur. Geranic acid was added directly or from ethanolic stock solutions at the beginning of the exponential growth phase (after 1 h of cultivation for *E. coli* and *P. putida* and after 3 h for *S. cerevisiae*). Controls with the highest applied ethanol concentrations were tested simultaneously and no inhibiting effects of ethanol on microbial growth could be observed. Samples were taken during exponential growth phase every 30 min for *E. coli* and *P. putida* and every hour for *S. cerevisiae* and OD_600_ was determined. All experiments were performed in duplicates. For each growth curve, μ_max_ was determined at the exponential growth phase immediately after geranic acid addition and divided by μ_max0_ of the culture without geranic acid.

For metabolization tests with geraniol as sole carbon source, *P. putida* cells were grown in complex medium overnight and plated on E2 minimal medium lacking glycerol. Geraniol was applied as vapor phase. Water was used as negative control and *p*-cymene as positive control, since *P. putida* DSM 12264 is known to grow on this aromatic compound [25]. Cell growth was monitored for 94 h at 30°C. For metabolization tests with geranic acid as the sole carbon source, 20 mL E2 medium lacking glycerol with 20 mM geranic acid was inoculated using 1 mL of a *P. putida* overnight culture. No carbon source was applied for the negative control and 20 mM *p*-cymene was applied as positive control. Cell growth was monitored via OD_600_ determination for 144 h at 30°C.

Stability tests with geranic acid and whole cell biotransformation with geraniol were performed with *P. putida* cells in 50 mL and 20 mL E2 medium, respectively, containing corresponding terpenoid concentrations. Medium was inoculated with TB preculture of *P. putida* at an initial OD_600_ of 0.2. Geranic acid concentrations at different time points were determined by HPLC analysis.

### *De novo* geranic acid synthesis

All cultures of recombinant *P. putida* strains contained 30 mg/L kanamycin for plasmid selection. Precultures were inoculated from cryo cultures using 5 mL LB with antibiotic and incubated overnight at 30°C and 180 rpm shaking (2.5 cm displacement). Main cultures of 50 mL E2 medium (containing 10% LB) in 300 mL Erlenmeyer flasks were then inoculated using 0.5 mL of precultures. Main cultures were incubated at 30°C and 180 rpm shaking frequency (2.5 cm displacement). Gene expression was induced after 3 h with 0.2% (w/v) *L*-rhamnose. Samples were taken and stored at −20°C until sample preparation.

### HPLC analysis

To *P. putida* culture samples of 500 μL or 1 mL, 50 μL or 100 μL 1 M HCl were added, respectively, and analytes were extracted using 500 μL hexane containing 200 μM thymol as an internal standard. For bioconversion assays, 1 mM thymol was used. Samples were centrifuged (5 min, 18,000 g) and the organic phase was analyzed by HPLC, consisting of a diode array detector and a C-18 column (Alltech Alltima, C18, 5 μm, 250 × 4,6 mm; C18 Precolumn, Grace GmbH and Co. KG, Worms). Substances were separated isocratically using acetonitrile/acidified water (containing 0.5% (v/v) 3 M phosphoric acid) in a ratio of 55:45 (v/v) as mobile phase. Geraniol and thymol were monitored at 202 nm, geranic acid at 217 nm.

### Fed-batch fermentation

*De novo* geranic acid production in the bioreactor was performed using a DASGIP SR0700ODLS system (DASGIP, Jülich, Germany). 5 mL of LB precultures with 30 mg/L kanamycin were used to inoculate 500 mL E2 medium (containing 10% LB and 30 mg/L kanamycin). Main cultures were incubated for 24 h at 30°C with 50 mL/h aeration and 30% saturation of dissolved oxygen (DO). Glycerol (612 g/L) and MgSO_4_ (10 g/L) feed was triggered by DO (50 mL/h feed rate). The pH was maintained at 6.9 by automatic addition of ammonia. GES expression was induced by addition of *L*-rhamnose after 24 h of incubation (t = 0) to a concentration of 0.2% (w/v) and aeration rate was lowered to 10 mL/h, DO limited to a saturation of 10% and a feed rates of 10 mL/h (glycerol and MgSO_4_) for the production phase. Samples were taken over time and prepared for HPLC analysis as described above.

## Additional files

Additional file 1:
**Statistical analysis of the dataset of biotransformation of geraniol to geranic acid by wildtype** ***P. putida*** **DSM 12264 (Figure** **2** **).**
Additional file 2:
**Schematic of the cloning procedure leading to the plasmids pMiS1, pMiS1-ges, pUC18-mva-op, pMiS1-hmgs, pMiS1-mva and pMiS1-ges-mva, respectively.**
Additional file 3:
**Statistical analysis of the dataset of** ***de novo*** **production of geranic acid by** ***P. putida*** **DSM 12264 harboring** ***ges*** **(Figure** **3** **).**
Additional file 4:
**Statistical analysis of the maximum concentration of geranic acid produced by** ***P. putida*** **DSM 12264 harboring** ***ges*** **and by** ***P. putida*** **DSM 12264 harboring** ***ges*** **and** ***mva*** **(Figures** **3** **and** **4** **).**

## Abbreviations

GES: Geraniol synthase
MVA: Mevalonate
OD_600_: Optical density at 600 nm
GPP: Geranyl pyrophosphate
FPP: Farnesyl pyrophosphate
IPP: Isopentenyl pyrophosphate
DMAPP: Dimethylallyl pyrophosphate
HPLC: High pressure liquid chromatography
DO: Dissolved oxygen
