# Supplementary material for: De novo production of the monoterpenoid geranic acid by metabolically engineered Pseudomonas putida
Source: Microb Cell Fact. 2014 Dec 4;13:170. doi: 10.1186/s12934-014-0170-8 (PMC4266966; doi:10.1186/s12934-014-0170-8)
Supplement: Additional file 1: — Statistical analysis of the dataset of biotransformation of geraniol to geranic acid by wildtype P. putida DSM 12264 (Figure 2 ). [file 12934_2014_170_MOESM1_ESM.pdf]

Additional file 1: Statistical analysis of the dataset of biotransformation of geraniol to geranic acid by wildtype *P. putida* DSM 12264 (Figure 2). ANOVA **(a)** and Tukey's HSD **(b-f)** for 4 mM, 8 mM, 16 mM, 32 mM and 64 mM geraniol as the initial substrate concentration were determined for the time points t = 8 h **(a/b)**, 16 h **(a/c)**, 32 h **(a/d)**, 38 h **(a/e)** and 64 h **(a/f)**, if values were available. Values of product concentrations were not considered, when no further formation of geranic acid was possible due to geraniol depletion. *: mean difference is significant at level $p < 0.05$ (Tukey's HSD).

**a) ANOVA**		**Sum of squares**	**df**	**Mean square**	**F**	**Sig.**
**t = 8 h**	**Between Groups**	3.24	4	.81	18.45	.000
	**Within Groups**	.44	10	.04		
	**Total**	3.68	14			
**t = 16 h**	**Between Groups**	5.12	3	1.71	5.95	.020
	**Within Groups**	2.29	8	.29		
	**Total**	7.41	11			
**t = 32 h**	**Between Groups**	19.28	2	9.64	7.3	.025
	**Within Groups**	7.93	6	1.32		
	**Total**	27.2	8			
**t = 38 h**	**Between Groups**	5.43	1	5.43	2.19	.213
	**Within Groups**	9.92	4	2.48		
	**Total**	15.34	5			
**t = 64 h**	**Between Groups**	9.48	1	9.48	1.61	.274
	**Within Groups**	23.6	4	5.9		
	**Total**	33.08	5			

b) TUKEY HSD t = 8 h

Applied geraniol concentration (I)	Applied geraniol concentration (J)	Mean difference (I-J)	Std. error	Sig.	95% Confidence interval	
					Lower bound	Upper bound
4 mM	8 mM	.04	.17	.999	-.52	.60
	16 mM	.09	.17	.984	-.47	.65
	32 mM	.71*	.17	.013	.15	1.27
	64 mM	1.17*	.17	.000	.61	1.74
8 mM	4 mM	-.04	.17	.999	-.60	.52
	16 mM	.05	.17	.998	-.51	.61
	32 mM	.67*	.17	.019	.11	1.23
	64 mM	1.13*	.17	.000	.57	1.70
16 mM	4 mM	-.09	.17	.984	-.65	.47
	8 mM	-.05	.17	.998	-.61	.51
	32 mM	.62*	.17	.030	.06	1.18
	64 mM	1.08*	.17	.001	.52	1.65
32 mM	4 mM	-.71*	.17	.013	-1.27	-.15
	8 mM	-.67*	.17	.019	-1.23	-.11
	16 mM	-.62*	.17	.030	-1.18	-.06
	64 mM	.46	.17	.122	-.10	1.03
64 mM	4 mM	-1.17*	.17	.000	-1.74	-.61
	8 mM	-1.13*	.17	.000	-1.70	-.57
	16 mM	-1.08*	.17	.001	-1.65	-.52
	32 mM	-.46	.17	.122	-1.03	.10

c) TUKEY HSD t = 16 h

Applied geraniol concentration (I)	Applied geraniol concentration (J)	Mean difference (I-J)	Std. error	Sig.	95% Confidence interval	
					Lower bound	Upper bound
8 mM	16 mM	-.45	.44	.732	-1.85	.94
	32 mM	.37	.44	.826	-1.03	1.77
	64 mM	1.32	.44	.065	-.08	2.72
16 mM	8 mM	.45	.44	.732	-.94	1.85
	32 mM	.83	.44	.301	-.57	2.23
	64 mM	1.77*	.44	.015	.38	3.17
32 mM	8 mM	-.37	.44	.826	-1.77	1.03
	16 mM	-.83	.44	.301	-2.23	.57
	64 mM	.95	.44	.213	-.45	2.34
64 mM	8 mM	-1.32	.44	.065	-2.72	.08
	16 mM	-1.77*	.44	.015	-3.17	-.38
	32 mM	-.95	.44	.213	-2.34	.45

d) TUKEY HSD t = 32 h

Applied geraniol concentration (I)	Applied geraniol concentration (J)	Mean difference (I-J)	Std. error	Sig.	95% Confidence interval	
					Lower bound	Upper bound
16 mM	32 mM	2.05	.94	.154	-.83	4.93
	64 mM	3.57*	.94	.021	.69	6.45
32 mM	16 mM	-2.05	.94	.154	-4.93	.83
	64 mM	1.53	.94	.306	-1.35	4.41
64 mM	16 mM	-3.57*	.94	.021	-6.45	-.69
	32 mM	-1.53	.94	.306	-4.41	1.35

e) TUKEY HSD t = 38 h

Applied geraniol concentration (I)	Applied geraniol concentration (J)	Mean difference (I-J)	Std. error	Sig.	95% Confidence interval	
					Lower bound	Upper bound
32 mM	64 mM	1.90	1.29	.213	-1.67	5.47
64 mM	32 mM	-1.90	1.29	.213	-5.47	1.67

f) TUKEY HSD t = 64 h

Applied geraniol concentration (I)	Applied geraniol concentration (J)	Mean difference (I-J)	Std. error	Sig.	95% Confidence interval	
					Lower bound	Upper bound
32 mM	64 mM	2.51	1.98	.274	-2.99	8.02
64 mM	32 mM	-2.51	1.98	.274	-8.02	2.99
