# Supplementary material for: De novo production of the monoterpenoid geranic acid by metabolically engineered Pseudomonas putida
Source: Microb Cell Fact. 2014 Dec 4;13:170. doi: 10.1186/s12934-014-0170-8 (PMC4266966; doi:10.1186/s12934-014-0170-8)
Supplement: Additional file 2: — Schematic of the cloning procedure leading to the plasmids pMiS1, pMiS1-ges, pUC18-mva-op, pMiS1-hmgs, pMiS1-mva and pMiS1-ges-mva, respectively. [file 12934_2014_170_MOESM2_ESM.pdf]

Construction of pMiS1-ges

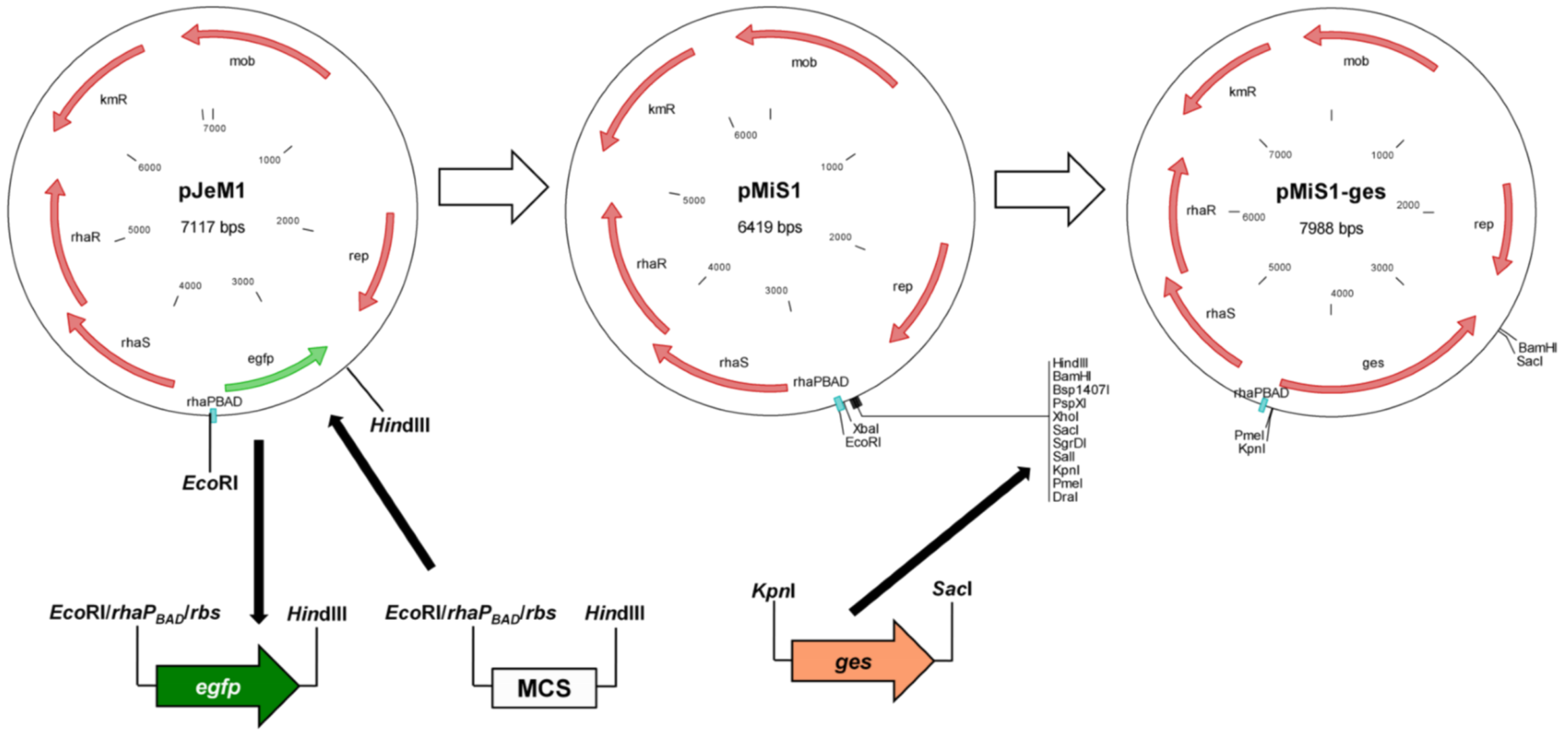

Additional file 2: Construction of pMiS1 and pMiS1-ges. pMiS1 was constructed by exchange of eGFP (enhanced green fluorescent protein) gene of pJeM1 (Jeske, M. and Altenbuchner, J., "The *Escherichia coli* rhamnose promoter *rhaP_{BAD}* is in *Pseudomonas putida* KT2440 independent of Crp–cAMP activation", Appl Microbiol Biotechnol (2010) 85:1923–1933) with a synthetically constructed MCS (multiple cloning site). Since the original rhamnose-inducible promoter *rhaP*$_{BAD}$ and the ribosome binding site were cut out with *egfp* simultaneously using *Eco*RI, *rhaP*$_{BAD}$ and a ribosome binding site were introduced with the MCS. Geraniol synthase gene *ges* of *Ocimum basilicum* was then introduced using *Kpn*I and *Sac*I to give pMiS1-ges. *rep*: gene required for plasmid replication, *mob*: gene required for plasmid mobilization, *kmR*: kanamycin-resistance gene, *rhaR*: activator gene, *rhaS*: activator gene, *rhaP*$_{BAD}$: rhamnose-inducible promoter region (blue box).

Construction of pUC18-mva-op

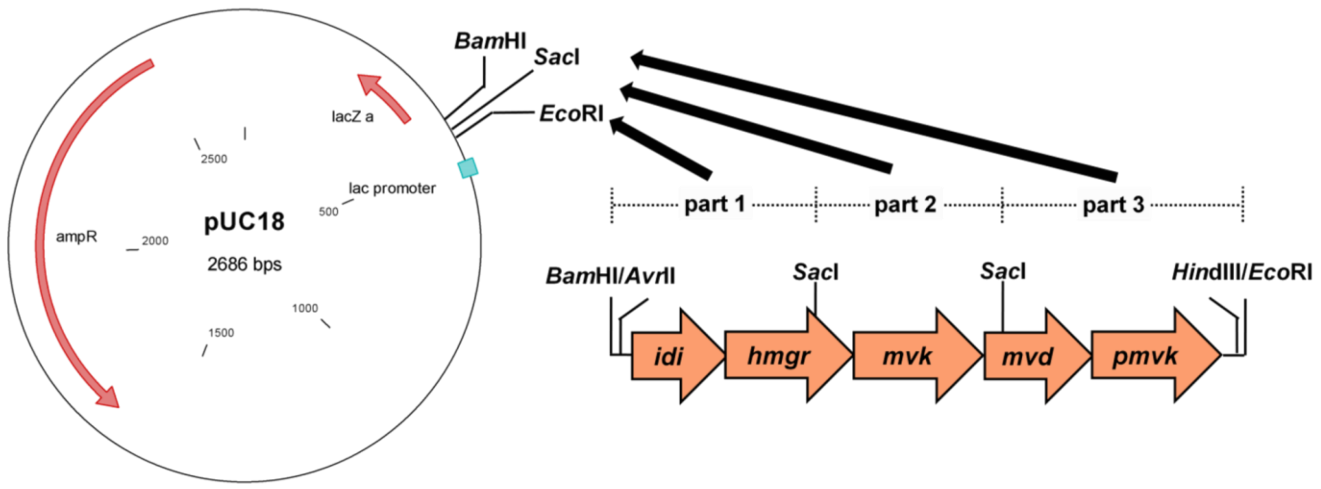

Additional file 4b: Construction of pUC18-mva-op. 5 of the 6 genes of the MVA pathway of *M. xanthus*, namely *idi*, *hmgr*, *mvk*, *mvd* and *pmvk*, are organized in an operon and were sequentially introduced into pUC18 (part 1, 2 and 3) using *Bam*HI, *Sac*I and *Eco*RI. Primers were designed to introduce an *Avr*II restriction site and a ribosome binding site upstream of *idi* and a *Hind*III restriction site downstream of *pmvk*. Blue box: *lac* promoter, *ampR*: ampicillin-resistence gene, *lacZ a*: gene for the alpha-polypeptide of beta-galactosidase.

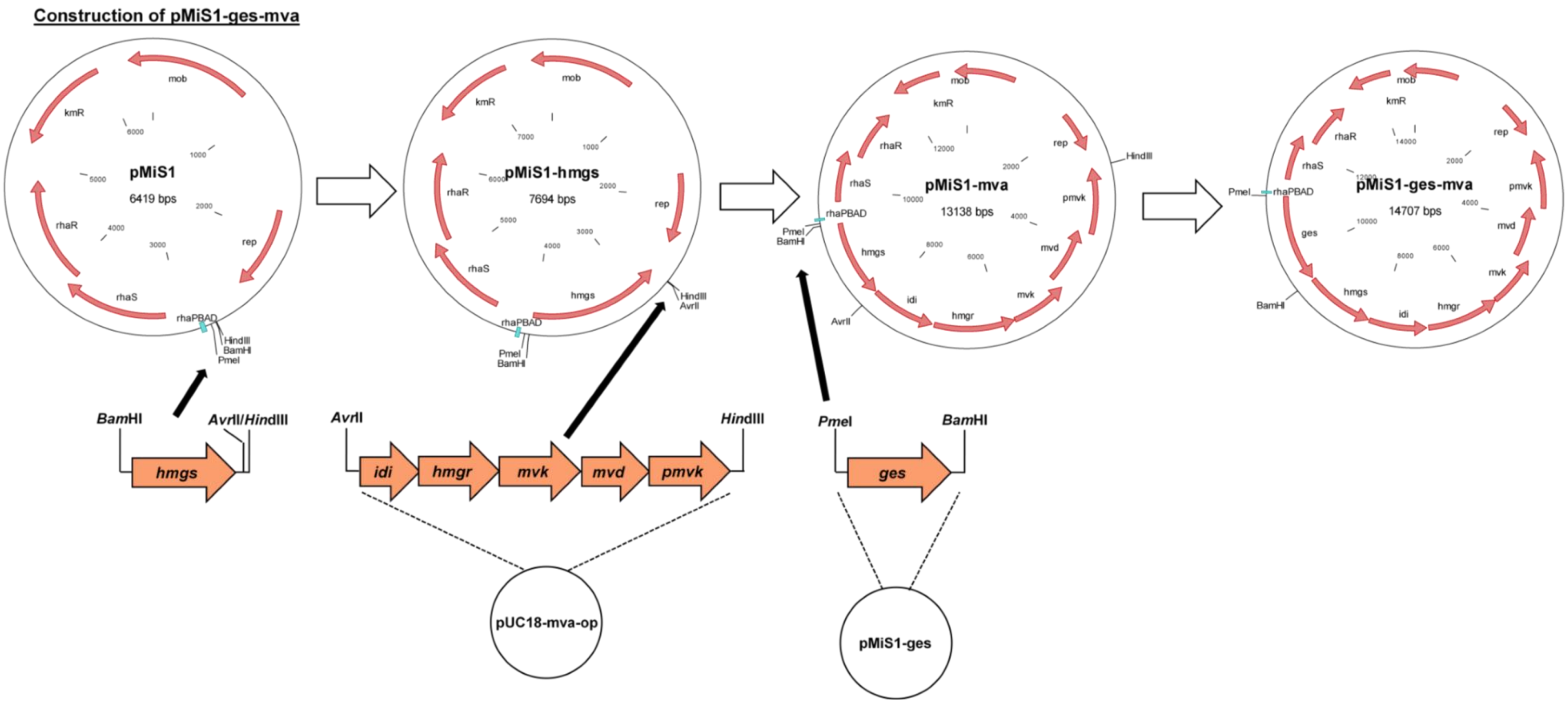

Additional file 4c: Construction of pMiS1-ges-mva. *hmgs* of *M. xanthus* was cloned into pMiS1 using *Bam*HI and *Hin*dIII to give pMiS1-hmgs. Primers were designed to add a ribosome binding site upstream and an *Avr*II restriction site downstream of the gene. The remaining 5 of the 6 genes of the MVA pathway of *M. xanthus* were then cut from pUC18-mva-op and introduced via *Avr*II and *Hin*dIII into pMiS1-hmgs to give pMiS1-mva. *ges* of pMiS1-ges was then introduced via *Pme*I and *Bam*HI to give pMiS1-ges-mva. *rep*: gene required for plasmid replication, *mob*: gene required for plasmid mobilization, *kmR*: kanamycin-resistance gene, *rhaR*: activator gene, *rhaS*: activator gene, *rhaP*$_{BAD}$: rhamnose-inducible promoter region (blue box).
