# Supplementary material for: De novo production of the monoterpenoid geranic acid by metabolically engineered Pseudomonas putida
Source: Microb Cell Fact. 2014 Dec 4;13:170. doi: 10.1186/s12934-014-0170-8 (PMC4266966; doi:10.1186/s12934-014-0170-8)
Supplement: Additional file 3: — Statistical analysis of the dataset of de novo production of geranic acid by P. putida DSM 12264 harboring ges (Figure 3 ). [file 12934_2014_170_MOESM3_ESM.pdf]

Additional file 3: Statistical analysis of the dataset of *de novo* production of geranic acid by *P. putida* DSM 12264 harboring *ges* (Figure 3). In table **a)** and **b)**, ANOVA and Tukey's HSD values are shown for t = 0 - t = 26.25 in regard to geraniol production by *P. putida* DSM 12264 w/o *ges* and *P. putida* DSM 12264 harboring *ges*. In table **c)** and **d)**, ANOVA and Tukey's HSD values are shown for t = 0 to t = 26.25 in regard to geranic acid production by *P. putida* DSM 12264 w/o *ges* and *P. putida* DSM 12264 harboring *ges*. *: mean difference is significant at level p < 0.05 (Tukey's HSD).

**a) ANOVA (geraniol)**		**Sum of squares**	**df**	**Mean square**	**F**	**Sig.**
**t = 0**	**Between Groups**	.00	1	.00	2.79	.237
	**Within Groups**	.00	2	.00		
	**Total**	.00	3			
**t = 4**	**Between Groups**	.30	1	.30	151.25	.007
	**Within Groups**	.00	2	.00		
	**Total**	.31	3			
**t = 5**	**Between Groups**	.36	1	.36	450.00	.002
	**Within Groups**	.00	2	.00		
	**Total**	.36	3			
**t = 10**	**Between Groups**	.12	1	.12	1156.00	.001
	**Within Groups**	.00	2	.00		
	**Total**	.12	3			
**t = 21.25**	**Between Groups**	.00	1	.00	NaN	NaN
	**Within Groups**	.00	2	.00		
	**Total**	.00	3			
**t = 22.25**	**Between Groups**	.00	1	.00	NaN	NaN
	**Within Groups**	.00	2	.00		
	**Total**	.00	3			
**t = 24.25**	**Between Groups**	.00	1	.00	NaN	NaN
	**Within Groups**	.00	2	.00		
	**Total**	.00	3			
**t = 26.25**	**Between Groups**	.00	1	.00	NaN	NaN
	**Within Groups**	.00	2	.00		
	**Total**	.00	3			

b) TUKEY HSD (geraniol)

	Strain (I)	Strain (J)	Mean difference (I-J)	Std. error	Sig.	95% Confidence interval<br>Lower bound	Upper bound
**t = 0**	control (w/o ges)	+ ges	-.05	.03	.237	-.16	.07
	+ ges	control (w/o ges)	.05	.03	.237	-.07	.16
**t = 4**	control (w/o ges)	+ ges	-.55*	.04	.006	-.74	-.36
	+ ges	control (w/o ges)	.55*	.04	.006	.36	.74
**t = 5**	control (w/o ges)	+ ges	-.60*	.03	.002	-.72	-.48
	+ ges	control (w/o ges)	.60*	.03	.002	.48	.72
**t = 10**	control (w/o ges)	+ ges	-.34*	.01	.000	-.38	-.30
	+ ges	control (w/o ges)	.34*	.01	.000	.30	.38
**t = 21.25**	control (w/o ges)	+ ges	.00	.00		.00	.00
	+ ges	control (w/o ges)	.00	.00		.00	.00
**t = 22.25**	control (w/o ges)	+ ges	.00	.00		.00	.00
	+ ges	control (w/o ges)	.00	.00		.00	.00
**t = 24.25**	control (w/o ges)	+ ges	.00	.00		.00	.00
	+ ges	control (w/o ges)	.00	.00		.00	.00
**t = 26.25**	control (w/o ges)	+ ges	.00	.00		.00	.00
	+ ges	control (w/o ges)	.00	.00		.00	.00

c) ANOVA (geranic acid)

		Sum of squares	df	Mean square	F	Sig.
**t = 0**	**Between Groups**	.00	1	.00	NaN	NaN
	**Within Groups**	.00	2	.00		
**Total**		.00	3			
**t = 4**	**Between Groups**	.00	1	.00	.16	.728

	**Within Groups**	.00	2	.00		
	**Total**	.01	3			
**t = 5**	**Between Groups**	.01	1	.01	.78	.471
	**Within Groups**	.03	2	.01		
	**Total**	.04	3			
**t = 10**	**Between Groups**	3.01	1	3.01	91.71	.011
	**Within Groups**	.07	2	.03		
	**Total**	3.08	3			
**t = 21.25**	**Between Groups**	62.25	1	62.25	421.76	.002
	**Within Groups**	.30	2	.15		
	**Total**	62.55	3			
**t = 22.25**	**Between Groups**	59.83	1	59.83	2587.25	.000
	**Within Groups**	.05	2	.02		
	**Total**	59.88	3			
**t = 24.25**	**Between Groups**	58.52	1	58.52	514.94	.002
	**Within Groups**	.23	2	.11		
	**Total**	58.75	3			
**t = 26.25**	**Between Groups**	61.07	1	61.07	564.33	.002
	**Within Groups**	.22	2	.11		
	**Total**	61.29	3			

d) TUKEY HSD (geranic acid)

						**95% Confidence interval**	
	**Strain (I)**	**Strain (J)**	**Mean difference (I-J)**	**Std. error**	**Sig.**	**Lower bound**	**Upper bound**
**t = 0**	control (w/o ges)	+ ges	.00	.00		.00	.00

	+ ges	control (w/o ges)	.00	.00		.00	.00
**t = 4**	control (w/o ges)	+ ges	-.02	.05	.728	-.23	.19
	+ ges	control (w/o ges)	.02	.05	.728	-.19	.23
**t = 5**	control (w/o ges)	+ ges	-.10	.11	.471	-.59	.39
	+ ges	control (w/o ges)	.10	.11	.471	-.39	.59
**t = 10**	control (w/o ges)	+ ges	-1.73*	.18	.011	-2.51	-.96
	+ ges	control (w/o ges)	1.73*	.18	.011	.96	2.51
**t = 21.25**	control (w/o ges)	+ ges	-7.89*	.38	.002	-9.54	-6.24
	+ ges	control (w/o ges)	7.89*	.38	.002	6.24	9.54
**t = 22.25**	control (w/o ges)	+ ges	-7.73*	.15	.000	-8.39	-7.08
	+ ges	control (w/o ges)	7.73*	.15	.000	7.08	8.39
**t = 24.25**	control (w/o ges)	+ ges	-7.65*	.34	.001	-9.10	-6.20
	+ ges	control (w/o ges)	7.65*	.34	.001	6.20	9.10
**t = 26.25**	control (w/o ges)	+ ges	-7.81*	.33	.001	-9.23	-6.40
	+ ges	control (w/o ges)	7.81*	.33	.001	6.40	9.23
