# Supplementary material for: De novo production of the monoterpenoid geranic acid by metabolically engineered Pseudomonas putida
Source: Microb Cell Fact. 2014 Dec 4;13:170. doi: 10.1186/s12934-014-0170-8 (PMC4266966; doi:10.1186/s12934-014-0170-8)
Supplement: Additional file 4: — Statistical analysis of the maximum concentration of geranic acid produced by P. putida DSM 12264 harboring ges and by P. putida DSM 12264 harboring ges and mva (Figures 3 and 4 ). [file 12934_2014_170_MOESM4_ESM.pdf]

Additional file 4: Statistical analysis of the maximum concentration of geranic acid produced by *P. putida* DSM 12264 harboring *ges* and by *P. putida* DSM 12264 harboring *ges* and *mva* (Figure 3 and 4). ANOVA **(a)** and Tukey's HSD **(b)** values of the maximum concentration of *de novo* geranic acid produced by *P. putida* DSM 12264 harboring *ges* and *P. putida* DSM 12264 harboring *ges* and *mva* are shown. *: mean difference is significant at level $p < 0.05$ (Tukey's HSD).

**a) ANOVA**	**Sum of squares**	**df**	**Mean square**	**F**	**Sig.**
**Between Groups**	42737.29	1	42737.29	177.00	.006
**Within Groups**	482.91	2	241.46		
**Total**	43220.20	3			

b) TUKEY HSD

					**95 Confide**	**% nce interval**
**Strain (I)**	**Strain (J)**	**Mean difference (I-J)**	**Std. error**	**Sig.**	**Lower bound**	**Upper bound**
+ges, w/o mva	+ges, +mva	-206.73*	15.54	.005	-273.53	-139.93
+ges, +mva	+ges, w/o mva	206.73*	15.54	.005	139.93	273.53
